# Acute and sub acute toxicity study on Sangu parpam

**DOI:** 10.6026/97320630017046

**Published:** 2021-01-31

**Authors:** Ramachandran Madhavan, Sathish Adithya, Bupesh Giridharan, Moonandi Murugesan, Raja Lakshman Raja

**Affiliations:** 1 Department of Nanju Maruthuvam, National Institute of Siddha,Chennai, India; 2 Department of Sattam Sarntha Maruthuvamum Nanju Maruthuvamum, Sri Sairam Siddha Medical College and Research Centre, Chennai, India; 3 Department of Forest science Nagaland University (Central) Lumam, Zunheboto,India; 4 Research and Development Wing, Sree Balaji Medical College and Hospital, Chennai,BIHER, India

**Keywords:** Peptic ulcer, siddha, sangu parpam, anti ulcer activity

## Abstract

Peptic ulcer is described in the siddha system of medicinal classification of 4448 diseases. Information on the use of Sangu Parpam in treating peptic ulcer is known. Therefore, it is of interest to document the acute and sub acute toxicity analysis on Sangu parpam in this regard.

## Background

Traditional Medicine has played an important role in meeting the demands of primary health care in many developing countries and its use has expanded widely in many developed countries [1]. The rate of Peptic ulcers has increased [2]. Known treatments for peptic ulcer show many side effects like cardiac arrhythmias, hypertension and nephritis [3]. Information on the use of Sangu Parpam in treating peptic ulcer is known [4]. Therefore, it is of interest to document the acute and sub acute toxicity analysis on Sangu parpam in this regard.

## Methodology

### Preparation of Sangu Parpam (SP):

Purification of Sangu:

Sangu was processed in Thaalithal method (Heating process) by covering it with Karchunnam (limestone) [5].

### Preparation process:

100g of purified Sangu from each purification process was covered by the grounded paste of Uthamani (Pergularia damea). This is kept in the mud lid and closed by another mud lid. Cotton ribbon soaked in wet clay was winded over the rims of both mud lids and let to dry in sun light for 8 hours. This set up was subjected to Ganapudam using 100 cow cakes. The set up was taken out after cooling. The calcinated Sangu was grounded well and stored in an airtight container [4].

### Anti ulcer studies:

Pylorus ligation method:

Albino wister rats of either sex weighing between 150 to 200gm were divided into six groups of 6 animals each as described below.

Group I: Control (Ghee 5ml/kg) 

Group II: Only pylorus ligation 

Group III: pylorus ligation + Ranitidine 30 mg/kg body weight, oral 

Group IV: pylorus ligation + SANGU PARPAM 9.36mg/200gm 

Group V: pylorus ligation + SANGU PARPAM 46.8mg/200gm 

Group VI: pylorus ligation + SANGU PARPAM 93.6mg/200gm 

The Albino Wister Rats were kept under fasting for 24 hours in metabolic cages without coprophagy (the eating of faeces). Three doses of SANGU PARPAM and the standard drug (Ranitidine 30 mg/kg) were given at different doses for five days orally [6]. The animals were kept under fasting for 14 hours with water ad libitum (as much or as often as necessary or desired) at the end of the 5th day. SANGU PARPAM was administered to the animals at about 30 minutes before the ligation. The abdomen was opened and pylorus ligated under mild ether anesthesia. The abdomen was sutured and care was taken to avoid bleeding or to occlude blood vessels. The animals were then sacrificed after 6 hours of pyloric ligation with surplus ketamine hydrochloride and the stomach was dissected. Gastric juice was collected from the sacrificed animal and its volume, pH, free acidity and total acidity was measured. Ulcer index was then determined. Evaluation of antioxidant enzymes such as SOD, CAT, lipidperoxidation, Myeloperoxidation, and Histopathological evaluation were completed from the excised stomach".

### Ethanol/HCL induced ulcer method:

Albino Wister rats were divided into 6 groups of 6 animals each. The animals were of either sex and were of nearly 150-200g in weight as described below.

Group I: Control (Ghee 5 ml/kg)

Group II: Negative Control (Hcl/Ethanol mixture containing 0.15 N Hcl in 70% v/v ethanol 1.5 ml) p.o

Group III: Hcl/Ethanol+ ranitidine 30 mg/Kg body weight, oral. 

Group IV: Hcl/Ethanol+ SANGU PARPAM 9.36mg/200g 

Group V: Hcl/Ethanol+ SANGU PARPAM 46.8mg/200g 

Group VI: Hcl/Ethanol+ SANGU PARPAM 93.6mg/200g 

The animals were kept under fasting for 24 hours with drinking water ad libitum until 2 hours before the start of the experiment [7]. Gastric injury was induced with acidified ethanol solution (150mM HCl/absolute ethanol) 40:60 v/v, (HCl/ethanol solution). Ghee was administered orally to the normal control groups and normal saline was administered to the ulcer control groups. 20mg/kg omeprazole was orally administered and for the experimental groups, oral administration of Sangu parpam 9.36mg, 46.8mg, and 93.6 mg/200g was given for the reference group. Ghee and normal saline was orally administered to the normal control group and ulcer control group, respectively after one hour of this pretreatment. The experimental group was administered with HCl/ ethanol solution (5ml/kg) orally for inducing gastric ulcers except normal control group. The rats were euthanized 60 minutes after the treatment with an excess of xylazine and ketamine anesthesia. The stomach was immediately excised and the ulcer index determined. The anti oxidant enzymes such as SOD, CAT, GPX, Lipid peroxidation and MPO were analyzed [8].

## Results:

The animals treated with all the dose levels did not produce any significant weight variations throughout the study period. The animals treated with SP at the dose of 9.36, 46.8 and 93.6mg/kg showed a statistically significant decrease (p < 0.05) in the free acidity level when compared to the normal control group (Table 1 - see PDF). The pyloric ligation group showed a marked increase in the total acidity level when compared to normal control group, which is statistically significant (p < 0.05). In animals treated with Sangu Parpam in different doses showed a statistically significant variation in gastric pH (p < 0.05) and total volume of gastric juice when compared to normal control animals (p < 0.05) (Table 2 - see PDF). The ulcer score as well as the ulcer index of the Sangu Parpam also showed a significant variation (P < 0.01) (Table 3 - see PDF) when compared with control group. There is no significant variation in the total protein (Table 4 - see PDF) level of the Sangu Parpam treated group with control group.

In ulcer-induced group the anti oxidant enzymes SOD, CAT, GPX, LPO and MPO were decreased when compared with control group. SP and Standard administered group shows increased in anti oxidant enzyme level there by protect Ulcer formation and also found to possess Anti ulcer activity (Tables 5 to 7 - see PDF). The ulcer score was found to increase in ethanol induced group of animals when compared with control group (p < 0.01). The ulcer index also showed a significant increase when compared with control groups (Table 8 - see PDF). In animals treated with SP in different doses showed a statistically significant decrease in Ulcer Score and Ulcer Index when compared with ethanol induced Ulcer group (p < 0.01) as shown in Table 9 (see PDF). The animals treated with Sangu parpam did not produce any significant variation in total protein level (Table 9 - see PDF). The SOD level was not significantly changed. Animals treated with 46.8mg/200g showed a significant increase (p < 0.01) in catalase and GPX levels while 93.6mg/200g group also showed a significant increase (p < 0.01). The LPO and MPO level did show significant variation (Tables 10 to 12 - see PDF). The animals treated with Sangu parpam and standard drug showed a significant increase in mucus weight (Table 13 - see PDF).

## Discussion:

**Figure 1:**
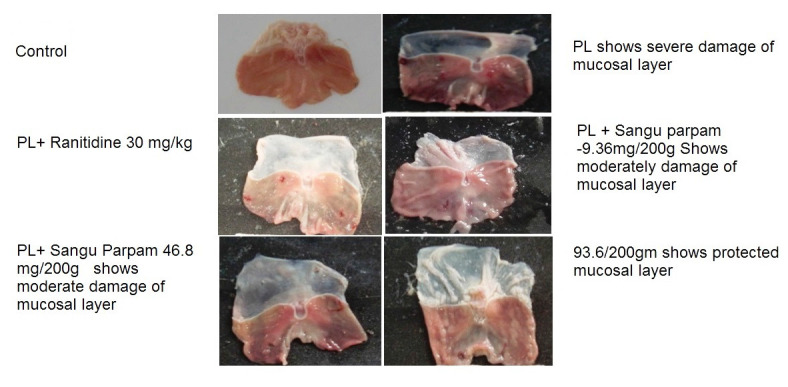
Macroscopic view of pylorus ligation (Pl) induced ulcer.

The Sangu parpam (SP) shows Anti Ulcer action in pyloric ligated rat models. The antiulcer property of Sangu parpam in pylorus ligation model is shown using significant reduction in free acidity, total acidity, number of ulcers and ulcer index [9]. Moreover, this suppressed the formation of ulcers. The inhibition of gastric ulcer in rats pre-treated with SP was comparable with ranitidine which is a standard drug used for curing gastric ulcer (Figure 1). Sangu parpam treated animals decreased both the concentration and increased the pH, and increased the gastric wall mucus, gastric mucosa. Thus, Sangu parpam suppress gastric damage induced by aggressive factors showing anti-ulcer activity.

**Figure 2:**
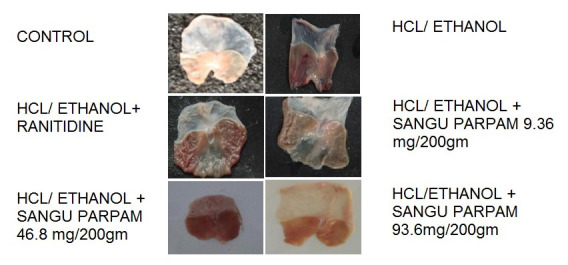
Macroscopic view of the gastric mucosa in HCl/Ethanol induced ulcer.

Peptic ulcers are caused by an imbalance between the protective and the aggressive mechanisms of the mucosa. The association of several endogenous factors and aggressive exogenous factors that are related to living conditions is shown. Sangu Parpam protects the gastric mucosa against Hcl-Ethanol induced injury on comparing the control group. The test drug shows significant increase in protection of gastric wall mucosa and also in ulcer area by inhibiting oedema and leukocyte infiltration of the sub mucosal area (Figure 2). A PGE2, SOD and CAT level of tissue homogenate reveals increased level of antioxidant enzymes in the treated group. Thus, this study shows that SP possesses an anti ulcer property.

## Conclusion

We document the acute and sub acute toxicity analysis on Sangu parpam in the context of treating peptic ulcer.
